# Spectrum and mortality of opportunistic infections among HIV/AIDS patients in southwestern China

**DOI:** 10.1007/s10096-022-04528-y

**Published:** 2022-11-21

**Authors:** Sirun Meng, Qiao Tang, Zhiman Xie, Nianning Wu, Yingmei Qin, Rongfeng Chen, Xiaoyu Chen, Xiu Chen, Yueqi Li, Minjuan Shi, Li Ye, Hao Liang, Junjun Jiang, Bo Zhou, Jianyan Lin

**Affiliations:** 1 The Fourth People’s Hospital of Nanning, Nanning, 530023 Guangxi China; 2 Guangxi Key Laboratory of AIDS Prevention and Treatment, School of Public Health, Guangxi Medical University, Nanning, 530021 Guangxi China; 3 Joint Laboratory for Emerging Infections Diseases in China (Guangxi)-ASEAN, Life Sciences Institute, Guangxi Medical University, Nanning, Guangxi China

**Keywords:** HIV/AIDS, Spectrum, Mortality, Opportunistic infections, Southwestern China

## Abstract

We describe the opportunistic infections (OIs) of HIV/AIDS to understand the spectrum, mortality, and frequency of multiple coinfected OIs among HIV/AIDS patients in southern China, where OIs are severe. We carried out a retrospective cohort study of hospitalized HIV-infected individuals at the Fourth People’s Hospital of Nanning, Guangxi, China, from Jan. 2011 to May. 2019. The chi-square test was used to analyze cross-infection; the Kaplan‒Meier analysis was used to compare mortality. A total of 12,612 HIV-infected patients were admitted to this cohort study. Among them, 8982 (71.2%) developed one or more OIs. The overall in-hospital mortality rate was 9.0%. Among the patients, 35.6% coinfected one OI, and 64.4% coinfected more than two OIs simultaneously. Almost half of the patients (60.6%) had CD4 + T-cell counts < 200 cells/μL. Pneumonia (39.8%), tuberculosis (35.3%), and candidiasis (28.8%) were the most common OIs. Coinfected cryptococcal meningitis and dermatitis are the most common combined OIs. The rate of anaemia (17.0%) was highest among those common HIV-associated complications. Multiple OIs are commonly found in hospitalized HIV/AIDS patients in southwestern China, which highlights the need for improved diagnosis and treatment.

## Introduction

AIDS has become a major global public health problem. The current discovery rate of HIV/AIDS is 75% globally, and in China, it is nearly 70%. The cases that have been found from a variety of people being tested included people who inject drugs, MSM, sex workers and their clients, and other traditional high-risk groups [1]. Globally, there were still 690,000 AIDS-related deaths in 2019 and 1.7 million new infections [2]. In 2019, there were 962,809 cases of AIDS reported in China and 316,477 AIDS-related deaths [3].

Individuals with chronic HIV infection not on treatment with antiretroviral agents, as the CD4 + T-cell counts drop, are vulnerable to a multitude of infections that rarely occur in an immunocompetent host, hence the term opportunistic infections (OIs) [4]. Opportunistic infection is an important criterion for HIV infection in the AIDS phase, and it is also a major adverse clinical event for HIV-infected patients, which seriously affects the prognosis of patients. Combination antiretroviral therapy reduced mortality and morbidity, but global evidence has shown that OIs grossly affect the health and quality of life of HIV-infected people by causing higher morbidity and mortality among those individuals [5–7]. Xiao J. et al. [8] reported that opportunistic infections were the most common causes of death in hospitalized patients infected with HIV. Previous studies have shown that Candida infection, tuberculosis (TB), *Pneumocystis jiroveci* pneumonia (PCP), and cytomegalovirus infection (CMV) are common OIs among HIV-infected patients in China [9].

Coinfection with multiple opportunistic infections at once is common in advanced HIV/AIDS. There are many articles on HIV patients coinfected with opportunistic infections, and several small studies have reported mortality of opportunistic infections among HIV/AIDS patients, but they mainly investigated the impact of HIV infection on the disease progression of opportunistic infections [8, 10–14], and it is unclear as to the incidence and distribution of multiple opportunistic infections or to what extent opportunistic infections influence the mortality and disease progression of individuals living with HIV/AIDS. This is especially true in Guangxi, southwestern China, where data on the prevalence of various OIs in admitted HIV-infected individuals are still lacking, and the spectrum of opportunistic infections and the risk of death from patients coinfected with different opportunistic infections are still unclear. In addition, multiple complications and opportunistic infections were another strong predictor of mortality in hospitalized HIV-infected patients.

The aim of our research was to understand the spectrum of opportunistic infections and mortality in hospitalized HIV-infected patients in Guangxi and to provide references for clinicians' treatment.

## Methods

### Study design and study population

This large-scale observational cohort study was conducted in the Fourth People’s Hospital of Nanning, the largest treatment center for HIV/AIDS in Guangxi Province, admitting more than 2500 HIV/AIDS patients each year. The present study included all HIV/AIDS patients admitted to the Fourth People’s Hospital of Nanning from January 2011 to May 2019. Individuals who were HIV/AIDS patients were identified by the hospital electronic medical record system. For those with multiple admissions, the latest admissions were preferentially included. HIV infection was determined by positive HIV ELISA and confirmatory Western blot assays. The mortality of inpatients was compared among HIV/AIDS patients with multiple opportunistic infections or without opportunistic infections. This study was approved by the Human Research Ethics Committee of Guangxi Medical University (Ethical Review No. 2019-SB-102).

### Definitions of various HIV-associated and non-HIV-associated opportunistic infections

The diagnosis of opportunistic infections and AIDS-defining malignancies was conducted on the basis of the guidelines for prevention and treatment of opportunistic infections in HIV-infected adults and adolescents recommended by the U.S. Centers for Disease Control and Prevention (CDC) [4, 15]. Opportunistic infections included pneumonia, tuberculosis, *Talaromyces marneffei*, candidiasis, hepatitis (B or C), syphilis, cytomegalovirus, herpesvirus, meningitis, and dermatitis. The diagnosis of pneumonia included bacterial pneumonia, viral pneumonia, pulmonary mycosis (including pneumocystis pneumonia), and pneumonia caused by other factors but did not include tuberculosis pneumonia, which was classified as tuberculosis [16]. Almost 100% of HIV-infected patients develop skin manifestations, including pruritus and pruritic skin rash, which manifest as nodular pruritus and specific dermatitis [17]. Dermatitis, including seborrheic dermatitis and atopic dermatitis, occurs early in the course of HIV disease and may be an initial clinical marker of HIV infection.

Diagnoses of HIV/AIDS and its opportunistic infections were also coinfected with medical history, clinical manifestations, X-ray imaging examinations, laboratory tests, and pathological data to perform comprehensive diagnosis.

Patients with other non-AIDS-related opportunistic infections and malignancies or with other internal diseases were defined as patients without opportunistic infections. HIV-associated complications, including hypertension, diabetes, tumour, respiratory failure, septicaemia, enteritis, hypoproteinaemia, electrolyte disturbances, anaemia, and immune reconstitution inflammatory syndrome, were diagnosed using the criteria in Internal Medicine.

### Data collection

Data for these individuals, including demographic characteristics, clinical and laboratory information, hospitalization time, outcomes of treatment, and CD4 + T-cell counts when admitted to the hospital, were obtained from the hospital electronic medical record system. The outcome at the time of hospital discharge was defined as (a) death or (b) survival. CD4 + T-cell counts when admitted to the hospital were categorized as (a) < 200 cells/μL, (b) 200–349 cells/μL, or (c) ≥ 350 cells/μL.

### Statistical analysis

The data were analyzed by using Statistical Package for the Social Sciences (SPSS, version 25.0) and R (version 4.0.3). The chi-square test and Fisher’s exact test were used for the proportional variable test. We used linear-by-linear association from the chi-square test for analysis of cross-infection between HIV/AIDS common opportunistic infections. The Kaplan‒Meier analysis was used to compare survival rates in groups with opportunistic infections. The log-rank test was used to assess significance. All mortality here refers to the mortality rate during hospitalization. A significance level of 0.05 was set for all statistical testing.

## Results

### Demographic and baseline clinical characteristics

**Fig. 1:**
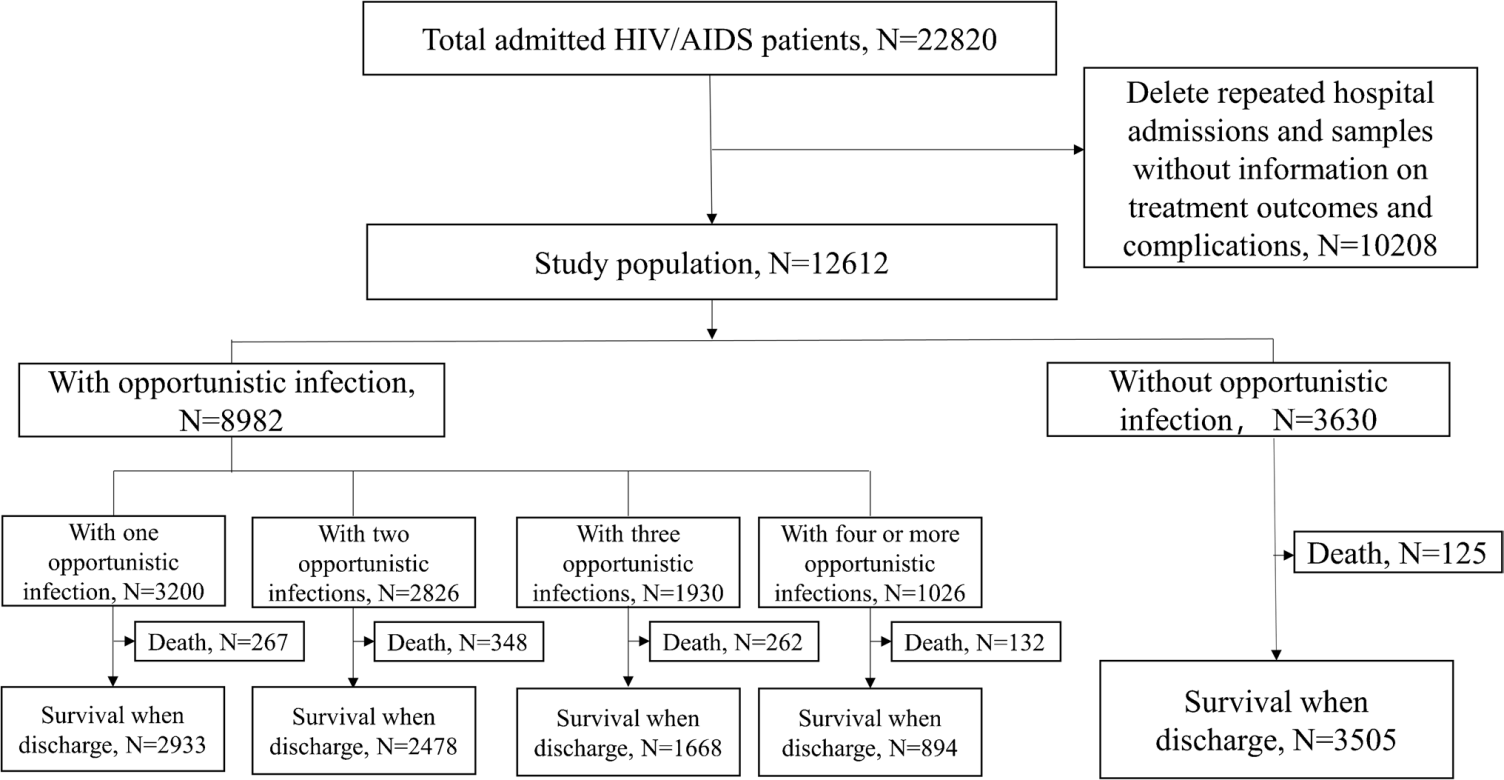
Flowchart of HIV/AIDS patients from 2011 to 2019 at Fourth People’s Hospital of Nanning, Guangxi, China

In this study, 22,820 HIV/AIDS patients admitted to the Fourth People’s Hospital of Nanning during January 2011 and May 2019 were screened. Among these 22,820 patients, complete clinical information was available in 12,612 (55.3%) and included in the analysis. Among them, 8982 (71.2%) were coinfected with opportunistic infections. Among the opportunistic infections patients, 3200 (35.6%) coinfected one opportunistic infection, 2826 (31.5%) coinfected two opportunistic infections, 1930 (21.5%) coinfected three opportunistic infections, and 1026 (11.4%) coinfected four or more opportunistic infections. Inpatients without opportunistic infections had the highest survival rate of 96.6%. The screening process is shown in Fig. 1.

The demographic characteristics of the 12,612 patients are shown in Table 1. In total, among the 12,612 HIV/AIDS patients, 9582 (76.0%) were males, and 3030 (24.0%) were females. A total of 5321 (42.2%) were aged 41–60 years, 3657 (29.0%) were aged over 60 years old, 7959 (63.1%) were married, 7780 (61.7%) were of Han nationality, 6737 (53.4%) were farmers, almost half of the patients with 60.6% (7644/12,612) had CD4 + T-cell counts < 200 cells/μL, and the most coinfected with opportunistic infections patients also had CD4 + T-cell counts < 200 cells/μL, accounting for 84.4% (6454/8982).

**Table 1:** Descriptive characteristics and opportunistic infection of HIV/AIDS patients from 2011 to 2019 at Fourth People’s Hospital of Nanning, Guangxi, China

Characteristics	Total patients, *n*	Percent (%)	Without opportunistic infection (%)	Coinfected with opportunistic infections (%)	*χ*^2^	*P*
Sex					104.351	< 0.001
Male	9582	76.0	2536 (26.5)	7046 (73.5)		
Female	3030	24.0	1094 (36.1)	1936 (63.9)		
Age (years)					11.023	0.012
< 20	232	1.8	72 (31.0)	160 (69.0)		
20–40	3402	27.0	937 (27.5)	2465 (72.5)		
41–60	5321	42.2	1497 (28.1)	3824 (71.9)		
> 60	3657	29.0	1124 (30.7)	2533 (69.3)		
Marital status					7.272	0.026
Married	7959	63.1	2342 (29.4)	5617 (70.6)		
Unmarried	2288	18.1	607 (26.5)	1681 (73.5)		
Other	2365	18.8	681 (28.8)	1684 (71.2)		
Nationality					10.474	0.005
Han	7780	61.7	2294 (29.5)	5486 (70.5)		
Zhuang	4492	35.6	1223 (27.2)	3269 (72.8)		
Other	340	2.7	113 (33.2)	227 (66.8)		
Occupation					40.078	< 0.001
Farmer	6737	53.4	1785 (26.5)	4952 (73.5)		
Unemployed	2303	18.3	693 (30.1)	1610 (69.9)		
Other	3572	28.3	1152 (32.3)	2420 (67.7)		
CD4 + T-cell counts when admitted to hospital (cell/μL)					1849.272	< 0.001
< 200	7644	60.6	1190 (15.6)	6454 (84.4)		
200–349	1992	15.8	884 (44.4)	1108 (55.6)		
≥ 350	2195	17.4	1285 (58.5)	910 (41.5)		
Missing	781	6.2	271 (34.7)	510 (65.3)		
In-hospital death					191.719	< 0.001
No	11,478	91.0	3505 (30.5)	7973 (69.5)		
Yes	1134	9.0	125 (11.0)	1009 (89.0)		

Sex, age, marital status, nationality, occupation, and CD4 + T-cell counts were significantly different between patients without opportunistic infections and those with opportunistic infections (*P* < 0.05).

### Opportunistic infection spectrum

Pneumonia (39.8%), tuberculosis (35.3%), and candidiasis (28.8%) were the most common opportunistic infections in these hospitalized patients, and patients with cryptococcal meningitis (23.8%), *Talaromyces marneffei* (16.0%), pneumonia (13.7%), or candidiasis (13.5%) had higher mortality. Tuberculosis is the most common disease among hospitalized patients with a single OI (Table 2).

**Table 2:** Opportunistic infection spectrum of HIV/AIDS patients from 2011 to 2019 at Fourth People’s Hospital of Nanning, Guangxi, China

Opportunistic infection	Total patients, *n*	Percent (%)	Death, *n* (%)	Coinfected one OI	Coinfected two OIs	Coinfected three OIs	Coinfected four or more OIs
Pneumonia	5016	39.8	687 (13.7)	955	1629	1507	925
Tuberculosis	4458	35.3	455 (10.2)	1016	1391	1252	799
*Talaromyces marneffei*	1765	14.0	282 (16.0)	147	474	474	611
Candidiasis	3633	28.8	489 (13.5)	358	1168	1276	831
Hepatitis (B or C)	1510	12.0	162 (10.7)	318	353	416	423
Syphilis	376	3.0	24 (6.4)	101	94	82	99
Cytomegalovirus	1087	8.6	107 (9.8)	81	223	362	421
Herpesvirus	305	2.4	13 (4.3)	108	88	53	56
Cryptococcal meningitis	269	2.1	64 (23.8)	24	86	77	82
Dermatitis	555	4.4	19 (3.4)	92	146	154	163

### Analysis of cross-infection between HIV/AIDS common opportunistic infections and the impact of cross-infection on mortality

Complication with cryptococcal meningitis and dermatitis (*χ*^2^ = 82.882, *P* < 0.001) are the most common opportunistic infections that cause the mortality of these HIV/AIDS hospitalized patients. Moreover, the coinfection risks of dermatitis were significantly higher than all the other HIV/AIDS common opportunistic infections in HIV/AIDS patients who were seropositive for pneumonia, *Talaromyces marneffei*, and candidiasis. In addition to pneumonia with candidiasis, tuberculosis with hepatitis (B or C) or cytomegalovirus, hepatitis (B or C) with cytomegalovirus, syphilis with herpesvirus, and herpesvirus with dermatitis cross-infection did not easily lead to death. Pairwise coinfection of other opportunistic infections increased the risk of mortality in these patients, and the difference was statistically significant (*P* < 0.05) (Table 3).

**Table 3:** Effects of opportunistic infections cross-infection on the mortality of HIV/AIDS patients from 2011 to 2019 at Fourth People’s Hospital of Nanning, Guangxi, China*

	Tuberculosis	*Talaromyces marneffei*	Candidiasis	Hepatitis (B or C)	Syphilis	Cytomegalovirus	Herpesvirus	Cryptococcal meningitis	Dermatitis
Pneumonia	27.113 *P* < 0.001	5.546 *P* = 0.019	0.100 *P* = 0.752	9.031 *P* = 0.003	16.339 *P* < 0.001	11.715 *P* = 0.001	22.393 *P* < 0.001	21.341 *P* < 0.001	47.642 *P* < 0.001
Tuberculosis		40.326 *P* < 0.001	20.558 *P* < 0.001	0.332 *P* = 0.353	5.677 *P* = 0.017	0.126 *P* = 0.722	11.381 *P* = 0.001	47.895 *P* < 0.001	35.050 *P* < 0.001
*Talaromyces marneffei*			6.148 *P* = 0.013	19.125 *P* < 0.001	23.282 *P* < 0.001	21.479 *P* < 0.001	29.193 *P* < 0.001	10.092 *P* = 0.001	58.911 *P* < 0.001
Candidiasis				7.197 *P* = 0.007	15.289 *P* < 0.001	9.916 *P* = 0.002	21.396 *P* < 0.001	21.974 *P* < 0.001	45.490 *P* < 0.001
Hepatitis (B or C)					6.391 *P* = 0.026	0.533 *P* = 0.465	12.171 *P* < 0.001	35.115 *P* < 0.001	27.069 *P* < 0.001
Syphilis						4.101 *P* = 0.043	1.472 *P* = 0.225	40.274 *P* < 0.001	4.452 *P* = 0.035
Cytomegalovirus							9.412 *P* = 0.002	38.040 *P* < 0.001	21.363 *P* < 0.001
Herpesvirus								46.854 *P* < 0.001	0.386 *P* = 0.534
Cryptococcal meningitis									82.882 *P* < 0.001

^*^Values correspond to *P* for trend and *P*-value

### Comparison of mortality between HIV patients with opportunistic infections and all common HIV-associated complications as well as CD4 + T-cell status

As Table 4 shows, the mortality rates of infectious shock and respiratory failure were the highest among the common HIV-related complications. The coinfection rate of anaemia (17.0%) was highest in those common HIV-associated complications, which included hypertension, diabetes, tumour, enteritis, respiratory failure, electrolyte disturbances, infectious shock, hypoproteinaemia, immune reconstitution inflammatory syndrome, diffuse massive bleeding, anaemia, and thrombocytopenia. Subgroup analysis of the situation of HIV patients showed that hypertension (38.5%) and diabetes (35.3%) were the highest HIV-associated complications in this population without opportunistic infection patients; tumour (38.5%) was the highest HIV-associated complication among those patients coinfected with one opportunistic infection; respiratory failure (35.8%, 25.7%) was the highest HIV-associated complication among those patients coinfected with two and three opportunistic infections; and thrombocytopenia (18.8%) was the highest HIV-associated complication among those patients coinfected with four or more opportunistic infections. Almost all patients (60.6%) coinfected with opportunistic infections had CD4 + *T* values below 200 cells/μL. Except for tumours, all the other common HIV-associated complications were significantly associated with opportunistic infection coinfection.

**Table 4:** Clinical indicators and other complications of HIV/AIDS patients with opportunistic infections from 2011 to 2019 at Fourth People’s Hospital of Nanning, Guangxi, China

Clinical indicators	Death, *n* (%)	Without OI (%)	Coinfected one OI (%)	Coinfected two OIs (%)	Coinfected three OIs (%)	Coinfected four or more OIs (%)	Total (%)	*χ*^2^	*P*
Hypertension	112 (12.3)	350 (38.5)	251 (27.6)	190 (20.9)	87 (9.6)	32 (3.5)	910 (7.2)	81.687	< 0.001
Diabetes	48 (11.1)	153 (35.3)	124 (28.6)	85 (19.6)	40 (9.2)	31 (7.2)	433 (3.4)	21.419	< 0.001
Tumour	15 (15.6)	25 (26.0)	37 (38.5)	16 (16.7)	12 (12.5)	6 (6.3)	96 (0.8)	9.206	0.056
Enteritis	72 (13.8)	69 (13.2)	126 (24.1)	145 (27.8)	114 (21.8)	68 (13.0)	522 (4.1)	84.384	< 0.001
Respiratory failure	277 (52.8)	34 (6.5)	102 (19.4)	188 (35.8)	135 (25.7)	66 (12.6)	525 (4.2)	198.296	< 0.001
Electrolyte disturbances	356 (22.4)	170 (10.7)	340 (21.4)	440 (27.7)	380 (23.9)	261 (16.4)	1591 (12.6)	481.716	< 0.001
Infectious shock	238 (76.5)	23 (7.4)	63 (20.3)	106 (34.1)	72 (23.2)	47 (15.1)	311 (2.5)	105.278	< 0.001
Hypoproteinemia	262 (22.5)	170 (14.6)	228 (19.6)	306 (26.2)	262 (22.5)	200 (17.2)	1166 (9.2)	287.170	< 0.001
Immune reconstitution inflammatory syndrome	11 (6.1)	4 (2.2)	52 (28.9)	60 (33.3)	41 (22.8)	23 (12.8)	180 (1.4)	66.880	< 0.001
Diffuse massive bleeding	11 (31.4)	19 (54.3)	9 (25.7)	5 (14.3)	2 (5.7)	0 (0.0)	35 (0.3)	13.930	0.008
Anaemia	333 (15.5)	455 (21.2)	426 (19.9)	534 (24.9)	430 (20.1)	298 (13.9)	2143 (17.0)	233.051	< 0.001
Thrombocytopenia	136 (24.1)	88 (15.6)	116 (20.6)	134 (23.8)	120 (21.3)	106 (18.8)	564 (4.5)	137.710	< 0.001
CD4 + T-cell counts < 200 when admitted to hospital (cells/μL)	878 (11.5)	1190 (15.6)	1798 (23.5)	2101 (27.5)	1628 (21.3)	927 (12.1)	7644 (60.6)	2262.742	< 0.001
Total	1134 (9.0)	3630 (28.8)	3200 (25.4)	2826 (22.4)	1930 (15.3)	1026 (8.1)	12,612		

## Discussion

In this study, we observed that most of the hospitalized HIV/AIDS patients (71.2%) were coinfected with opportunistic infections, and the overall in-hospital mortality rate was 9.0%. Inpatients with opportunistic infections had a higher mortality rate of 11.2%. Compared with other countries and other regions, the opportunistic infections and deaths of HIV patients in Guangxi are more serious and need to be improved. In the North American AIDS Cohort Collaboration on Research for HIV-infected persons in care during 2000–2010, rates of first OI were relatively low and generally declined over this time, of whom 5836 (9.0%) developed at least 1 OI [7]. In research in Korea, 24.4% of HIV-infected patients developed OIs [18]. Among the HIV patients reported in Taiwan from 2010 to 2014, 6413 (24.4%) PLWHA developed OIs [5]. Similar to our research, the number of Shanghai hospitalized HIV-infected patients due to OIs is still high, and 94.7% of the patients acquired OIs [19].

Analysis of the opportunistic infections spectrum found that pneumonia, tuberculosis, and candidiasis are the most common opportunistic infections in these hospitalized patients. Similar to many other papers, pneumonia and tuberculosis are the most common coinfected opportunistic infections in HIV patients. In addition, candidiasis, cytomegalovirus, cryptococcosis, and hepatitis (B or C) are also important life-threatening opportunistic infections [7–9, 11, 18]. Our study found that hospitalized patients with cryptococcal meningitis, *Talaromyces marneffei*, candidiasis, or pneumonia had a higher mortality rate. Previous studies have found that although tuberculosis is the main cause of death in HIV/AIDS patients in Guangxi, the number of *T. marneffei* coinfected patients has been increasing in recent years, and *T. marneffei* has become one of the main causes of death in HIV/AIDS patients [16]. Isolation of *T. marneffei* remains the gold standard for diagnosis [20]. In our study, the time to positivity for automated blood culture is around 7 days (range: 3–14 days) (personal observation). It cannot meet the urgent needs of rapid clinical diagnosis and early treatment, which may be the cause of its relatively high mortality rate.

In addition, we found in the analysis of the cross-infection of common opportunistic infections of HIV/AIDS and the impact of coinfection on mortality that coinfected cryptococcal meningitis and dermatitis are the most common combined opportunistic infections that cause the death of these HIV/AIDS hospitalized patients. The coinfection risks of dermatitis were significantly higher than all the other HIV/AIDS common opportunistic infections in HIV/AIDS patients who were seropositive for pneumonia, *Talaromyces marneffei* and candidiasis. Mucocutaneous manifestations such as oral candidiasis and seborrheic dermatitis are very common HIV-related opportunistic events and are usually initial markers of immunodeficiency [21]. That whether the immune dysregulation of HIV infection can induce atopic dermatitis is not proven, but it appears likely.

In the analysis of these inpatients, we found that HIV-related complications are also worthy of attention. The mortality rates of infectious shock and respiratory failure are the highest among the common HIV-related complications, but the coinfection rate of anaemia was highest. In Fujian, China, more than half of inpatients with HIV were anaemic, but severe anaemia is infrequent. Chinese HIV patients, especially those with TB, TM infection, and low CD4 levels, should be routinely detected for anaemia to improve therapy [22]. Electrolyte disturbances were the highest HIV-associated complication except anaemia in these coinfected opportunistic infections patients. HIV-infected patients, particularly those with AIDS, are predisposed to a host of different water, electrolyte, and acid–base disorders (sometimes with opposing effects) since they are exposed to infectious, inflammatory, oncological, and pharmacological variables whose combination undermines their homeostatic capability [23].

This study is limited because the research coverage is not very complete, and since some patients in northern Guangxi have not been included, it cannot fully represent the incidence in Guangxi. This is a retrospective database extraction, and its largest data are obtained from a single hospital. Therefore, some missing values and selection bias may affect the results.

Our study describes the incidence of opportunistic infections, disease spectrum, and severity of HIV-infected patients hospitalized in southwestern China and emphasizes the important contribution of opportunistic infections in increasing mortality in these HIV/AIDS patients. Our findings presented will assist clinicians in making a diagnosis and empirical treatment sooner and help generate a more successful HIV management strategy. Early diagnosis of OIs could significantly reduce associated mortality. In short, in HIV-infected patients, especially those with severely suppressed cellular immunity, opportunistic infections should be screened regularly for early diagnosis and early treatment to improve prognosis.

## Data Availability

Not applicable.

## Declarations

### Transparency declaration

The corresponding author had full access to all the data in the study and had final responsibility for the decision to submit for publication.
